# The Impact of Early Visual Deprivation on Spatial Hearing: A Comparison between Totally and Partially Visually Deprived Children

**DOI:** 10.3389/fpsyg.2017.00467

**Published:** 2017-04-10

**Authors:** Giulia Cappagli, Sara Finocchietti, Elena Cocchi, Monica Gori

**Affiliations:** ^1^Unit for Visually Impaired People, Istituto Italiano di TecnologiaGenova, Italy; ^2^Istituto David Chiossone per Ciechi ed IpovedentiGenova, Italy

**Keywords:** auditory perception, blindness, child development, spatial hearing, visual impairment

## Abstract

The specific role of early visual deprivation on spatial hearing is still unclear, mainly due to the difficulty of comparing similar spatial skills at different ages and to the difficulty in recruiting young blind children from birth. In this study, the effects of early visual deprivation on the development of auditory spatial localization have been assessed in a group of seven 3–5 years old children with congenital blindness (*n* = 2; light perception or no perception of light) or low vision (*n* = 5; visual acuity range 1.1–1.7 LogMAR), with the main aim to understand if visual experience is fundamental to the development of specific spatial skills. Our study led to three main findings: firstly, totally blind children performed overall more poorly compared sighted and low vision children in all the spatial tasks performed; secondly, low vision children performed equally or better than sighted children in the same auditory spatial tasks; thirdly, higher residual levels of visual acuity are positively correlated with better spatial performance in the dynamic condition of the auditory localization task indicating that the more residual vision the better spatial performance. These results suggest that early visual experience has an important role in the development of spatial cognition, even when the visual input during the critical period of visual calibration is partially degraded like in the case of low vision children. Overall these results shed light on the importance of early assessment of spatial impairments in visually impaired children and early intervention to prevent the risk of isolation and social exclusion.

## Introduction

The effects of early sensory deprivation on the acquisition of perceptual and cognitive skills have been extensively studied in order to measure the weight that each sensory modality has on the development of those skills (Bruner, 1959; Freedman, 1971; Mistretta and Bradley, 1978; Grubb and Thompson, 2004). Nonetheless, while the impact of early sensory loss on specific perceptual skills is usually easier to detect in adulthood when sensory systems have been already developed, the short-term as well as the long-term consequences of impaired perceptual functions in childhood are more difficult to predict. This is mainly due to the difficulty of comparing similar spatial skills at different ages and to the difficulty of assessing the spatial performance of congenitally and totally blind children at an early age. With this study, we aim to increase knowledge about the development of spatial hearing in visually impaired children by comparing spatial localization skills of totally blind and low vision participants in two tasks assessing the ability to encode static and dynamic sound sources separately.

Considering early visual deprivation, although it is often commonly accepted that the complete absence of vision from birth produces a strengthening of the remaining intact senses especially in adulthood (Collignon et al., 2009; Kupers and Ptito, 2014), a critical review of the available literature shows that non-visual modalities are not always able to fully compensate for the lack of visual experience, especially during childhood (Pasqualotto and Newell, 2007). In other words, although compensatory mechanisms for spatial perception have been demonstrated in blind individuals (Lessard et al., 1998; Voss et al., 2004; Doucet et al., 2005; Collignon and De Volder, 2009), an early and pervasive visual impairment might delay the development of specific auditory spatial skills (Zwiers et al., 2001; Lewald, 2002; Eimer, 2004; Gori et al., 2014; Cappagli et al., 2015; Kolarik et al., 2016). Neuroimaging studies tend to confirm the idea that the lack of vision sharpens the remaining modalities (James, 2013) by showing that the aforementioned superior abilities of blind individuals are supported by the recruitment of the otherwise unused visual cortex areas by the remaining non-visual modalities (Burton et al., 2002; Amedi et al., 2003; Noppeney et al., 2005; Collignon et al., 2009; Kupers et al., 2011; Thaler et al., 2011) that may have a functional role (Cohen et al., 1997; Amedi et al., 2004; Gougoux et al., 2005; Merabet et al., 2009). While the above studies suggest that non-visual sensory modalities can successfully compensate for the lack of vision, they don’t help to explain the poorer perceptual abilities reported by studies assessing spatial performance in blind individuals. Despite the effort in including all the results reported on the topic in a single comprehensive theory, to date the role of visual input in the development of spatial cognition is still unclear due to the high controversy of the results supporting the mentioned scientific hypotheses. The cross-sensory calibration hypothesis proposed by Gori et al. (2008, 2014) and supported by experimental data (Postma et al., 2008; Cappagli et al., 2015; Finocchietti et al., 2015; Vercillo et al., 2016) attempts to provide a comprehensive explanation by stating that during the early development vision calibrates other senses to process spatial information because vision is the most robust sense to perceive the spatial properties of the world. The spatial properties of the surrounding environment are indeed best discovered with vision because it provides an immediate and complete representation of multiple and simultaneous stimuli in the environment (Thinus-Blanc and Gaunet, 1997). As a consequence, the complete development of spatial cognition should be more compromised when a visual impairment occurs at an early age and is pervasive compared to when the visual impairment occurs later in life and affects only partially the visual status of the individual. The comparison between the effects of total blindness versus degraded vision on spatial perception at an early age provides experimental evidence concerning the essential role of visual experience in shaping space perception and cognition. For this reason, in our study we explicitly compared the performance of totally blind and low vision children in different spatial tasks and evaluated how the residual vision correlates with the spatial performance.

Moreover while the development of spatial cognition has been extensively studied in sighted children (Vasilyeva and Lourenco, 2012), it is not clear how visually impaired children represent the surrounding space by using the spatial maps constructed through the remaining intact senses, especially touch and hearing. Recent findings suggest that sighted children acquire spatial capabilities thanks to the reciprocal influence between visual perception and execution of movements (Bremner et al., 2008): children monitor the success of an action through a sensory-motor feedback by matching expected and observed changes of visual information. Indeed self-generated movements commonly help to perceive the space acoustically because they convey the proprioceptive sensation corresponding to the movement of the ears toward sound sources (Aytekin et al., 2008). Visually impaired children not only lack the visual input necessary to establish the sensory-motor feedback that typically promotes spatial development, but also manifest a general delay in the acquisition of important locomotor and proprioceptive skills which may cause them to accumulate much less spatial experience compared to their sighted peers (Fraiberg and Fraiberg, 1977; Warren, 1977; Landau et al., 1984). To perceive space, visually impaired children typically use hearing and touch. The case of hearing is particularly interesting because the auditory sense is not only the main channel for providing distal information (Spencer et al., 1989; Ungar, 2000) but also it might be superior to all other sensory alternatives because it provides spatial information in both active and passive conditions and it does not necessarily involve direct contact with objects (Wanet and Veraart, 1985; Jacobson, 1998). At the same time, the use of hearing to perceive distal information might be particularly difficult for visually impaired children because in this case they do not have any sensory feedback about sonorous objects in the far space. On the contrary, the haptic-proprioceptive system can provide accurate spatial data only within the scope of the body itself (Ungar, 2000), and therefore a blind person must actively move in the environment to sequentially touch all the stimuli embedded in the space. For this reason, we developed an auditory localization task which assesses the ability of children to localize by touch an auditory sound source in near space. Moreover, since it is not clear whether early visual deprivation differently impacts on the ability to localize static versus moving sound sources in adulthood (Lewald, 2013; Finocchietti et al., 2015), we employed two different tasks in which children were asked to indicate the spatial position of a static or dynamic sonorous stimulus. According to the cross-sensory calibration hypothesis proposed by Gori et al. (2008, 2014), we expected to observe a more evident spatial impairment in totally blind children, because low vision children could have benefit from the visual calibration of audition to encode spatial information. Since the available data on adults has not suggested whether we should expect a difference in the encoding of static and dynamic audio sound sources in early childhood, we didn’t postulate any specific hypothesis regarding the assessment of these skills in our sample.

## Materials and Methods

To increase knowledge about how the absence of vision can impact on the creation of spatial representations, and specifically for which spatial aspects the development of spatial cognition differs in blind and sighted children, we employed two auditory spatial tasks which assess the ability of children to localize a static (*Static Sound Localization Task*) or a moving (*Dynamic Sound Localization Task*) sound source. For all tasks, we compared the spatial performance of sighted and visually impaired groups of children.

### Task and Procedure

**FIGURE 1:**
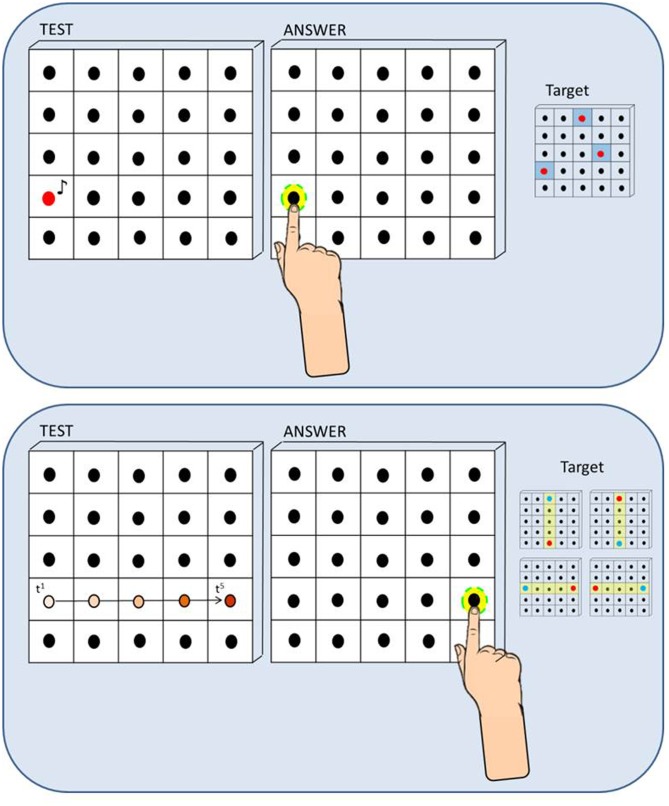
**Method and procedure. (A)** Static Sound Localization Task: In the Static Sound Localization Task, a sound coming from one out of three target loudspeakers (red dots on the right) was presented and the participant responded by touching the perceived sound source with the index finger of the dominant hand. Localization error was calculated for each trial by extracting the length of the vector that connected the actual and the perceived position of the loudspeaker (mm). **(B)** Dynamic Sound Localization Task: In the Dynamic Sound Localization Task, the sound moved across loudspeakers from a starting point (blue dots on the right) toward one of the four end-point positions (red dots on the right) and the participant responded by touching the end-point of the motion trajectory, that is the last active loudspeaker. Localization error was calculated for each trial by extracting the length of the vector that connected the actual and the perceived position of the loudspeaker (mm).

We developed a haptic setup made of a vertical surface (50 cm × 50 cm) covered by non-adjacent tactile sensors (2 cm × 2 cm) that can register the position of the contact and provide accurate information about spatial errors (**Figure 1**). The haptic setup consisted of 25 blocks of sensors, each block containing 16 tactile sensors (4 × 4) and a single loudspeaker in the middle. Therefore the total number of loudspeakers mounted on the setup was 25. The participant sat on a chair in front of a table which supported the haptic setup used to run the experiment. Distance from the setup to the trunk was maintained at 40 cm by positioning the chair in order to make the device easily reachable with the dominant hand for all the participants. While listening to the sound presented, the participant kept the dominant hand fixed on the starting point on the table that was approximately the position corresponding to the right limit of the haptic device (the left limit for left-handed).

The *Static Sound Localization Task* (**Figure 1**, upper panel) required the participant to indicate the position of a single sound source on a vertical surface. We selected three target loudspeakers sufficiently distributed on the surface (left, center, right) and judged equally difficult to reach in a previous pilot study: each target was sampled five times for a total of 15 trials. On each trial, a sound coming from one out of three target loudspeakers (red dots) was presented and the participant responded by touching the perceived sound source with the index finger of the dominant hand. The *Dynamic Sound Localization Task* (**Figure 1**, bottom panel) required the participant to indicate the end-point of a dynamic sound source that moved in the horizontal and vertical plane. We selected four motion trajectories centered on the setup (yellow rows: up-to-down, down-to-up, left-to-right, and right-to-left): each motion trajectory was sampled four times for a total of 16 trials. On each trial, the sound moved across loudspeakers from a starting point (blue dots) toward one of the four end-point positions (red dots) and the participant responded by touching the end-point of the motion trajectory, that is the last activated loudspeaker.

For both tasks, the auditory stimulus was a ‘meow’ sound registered and implemented in Matlab (R2013a, The MathWorks, USA). In the static condition of the task, a single sound was played at a time. In the dynamic condition of the task, while the first sound was playing the second sound started in order to create an audio motion. The sound has been chosen in order to make the task more entertaining for children. Children were instructed to listen the sound produced by the kitten and try to catch him. For both tasks, localization error was calculated for each trial by extracting the length of the vector that connected the actual and the perceived position of the loudspeaker (mm). Spatial accuracy indicated by localization error was computed for each participant and for each group of children.

### Participants

Fourteen sighted participants (mean age: 3.6, 10 males) and seven visually impaired participants (*N* = 5 low vision children, mean age: 4.4, 4 males; *N* = 2 blind children, mean age: 3.5, 2 males) have been enrolled in the study.

Visual acuity values are represented in LogMAR. The main exams used for the functional assessment of visual abilities are light sources method and Early Treatment Diabetic Retinopathy Study with Lea Hyvarinen symbols chart (Lea Symbols^®^ 15-Line Translucent ETDRS-Style Distance Chart). The distance from the chart was 3 m and assessment was performed with both eyes open using a backlit screen. The visual deficit of visually impaired participants has been interpreted according to the International Statistical Classification of Diseases and Related Health Problems (ICD) – 10th revision. The currently available version of the 10th revision of the International Statistical Classification of Diseases and Related Health Problems (ICD) defines visual impairment categories primarily on the basis of recommendations made by a World Health Organization (WHO) Study Group in 1972 (World Health Organization, 2003). The term ‘visual impairment’ in category H54 of the ICD classification, comprises category 0 for mild or no visual impairment, category 1 for moderate visual impairment, category 2 for severe visual impairment, categories 3, 4, and 5 for blindness, and category 9 for unqualified visual impairment. The term ‘low vision’ is used for visual acuity less than 0.5–1.3 LogMAR in the better eye with best correction and includes categories 1 and 2. The term ‘blindness’ is used for complete (no light perception) or nearly complete (visual acuity less than 1.3 LogMAR to light perception) vision loss. The participants in our study are defined as ‘low vision’ and ‘blind’ according to these definitions, except two children classified as ‘low vision’ who have a visual acuity of 1.7 LogMAR. In each case the visual deficit was of peripheral origin.

The cognitive level of all the visually impaired children was assessed with “The Reynell-Zinkin Scales: Developmental Scales for Young Visually Handicapped Children” and considered appropriate for their participation in the study, according to the total scores and the cut-offs proposed by the authors. Clinical details of the visually impaired children enrolled in the study can be found in **Table 1**. Sighted participants reported no visual impairment and a visual acuity better than 9/10. None of the sighted and visually impaired participants had additional sensory disabilities, including hearing disabilities tested with classical audiometer tests during the periodic neuroophthalmological assessment.

**Table 1:** Clinical details of visually impaired children.

Participant	Gender	Age	Visual status	Visual acuity (logMAR)	Pathology	Age at diagnosis
SI	M	3	Blind	Light perception	Retinopathy of prematurity (V)	Birth
S2	M	4	Blind	NPL	Bilateral anophthalmia	Birth
S3	M	5	Low vision	1.22	Bilateral coloboma	1 month
S4	M	5	Low vision	1.1	Leber hereditary optic neuropathy (LHON)	5 months
S5	M	4	Low vision	1.7	Osteopetrosis	Birth
S6	F	4	Low vision	1.22	Microphthalmus and coloboma (sx), Anophthalmia (dx)	Birth
S7	F	4	Low vision	1.7	Stargardt disease	4 months


*The table shows the clinical details for each visually impaired participant: visual status, visual acuity, pathology and age at diagnosis. Residual vision has been measured with different methods (light sources method, ETDRS Optotype and ERG, ERP, PEV) and is expressed in LogMAR. NPL (no perception of light)*.

Before entering the experimental room, all children with normal or residual vision were blindfolded so they had no chance to see the experimental setup. Before starting the test, each child was asked to familiarize with the experimental setup by exploring it with hands for 30 s. The study was approved by the ethics committee of the local health service and parental or adult informed written consent for the study was obtained in all cases.

## Results

**FIGURE 2:**
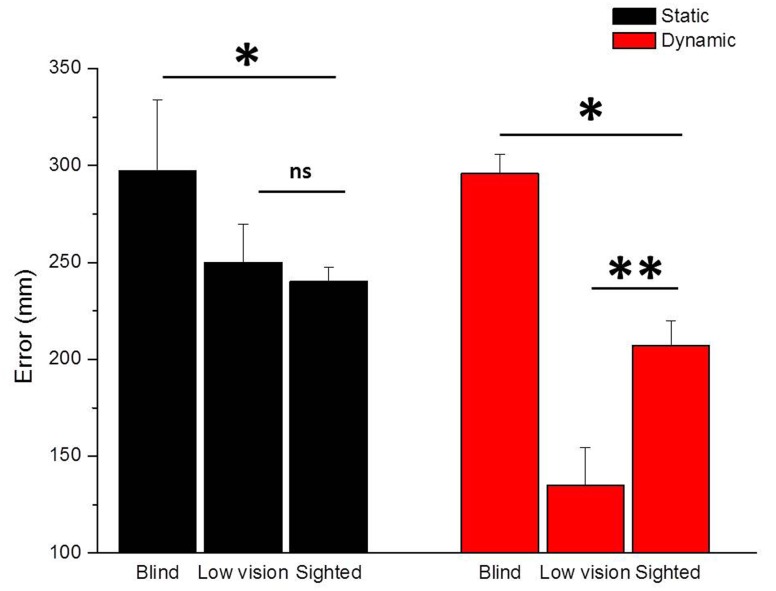
**Comparison between Static Sound Localization and Dynamic Sound Localization Tasks.** The figure shows the performance of blind and low vision children compared to sighted children for the Static Sound Localization Task (black bars) and the Dynamic Sound Localization Task (red bars). While for both tasks blind children performed significantly worse than sighted children (^∗^*p* < 0.05), low vision children performed better than sighted children only for the Dynamic Sound Localization Task (^∗∗^*p* < 0.01).

The average results of blind and low vision children are presented in **Figure 2** for the *Static Sound Localization Task* (black bars) and the *Dynamic Sound Localization Task* (red bars). As a measure of spatial accuracy, we plotted the localization error calculated as the average length of the vector that connected the correct and the perceived position of the loudspeaker (mm) for all trials and for each group.

When asked to localize a static sound source, totally blind children performed significantly worse than sighted children (*t* = -2.26, *p* < 0.05) but equally to low vision children (*t* = -1.68, *p* = 0.1). On the contrary, when asked to localize a dynamic sound source, blind children performed significantly worse compared to both sighted (*t* = -2.4, *p* < 0.05) and low vision (*t* = -4.28, *p* < 0.01) children. Moreover, low vision children showed a better performance than sighted children for the localization of a dynamic sound source (*t* = 4.94, *p* < 0.01) suggesting that early visual deprivation might impact differently the ability to localize fixed and moving auditory sound sources. To quantify the association between visual acuity and different aspects of spatial perception, we correlated the visual acuity expressed in LogMAR and the spatial accuracy expressed as mean localization error (mm) of all participants. The LogMAR scale is calculated as log (MAR) = log (1/V) = -log (V) and it represents vision loss, so higher values indicate poorer vision while lower values indicate better vision. The correlational analysis further confirmed our hypothesis, by indicating that a significant positive correlation exists when considering the dynamic sound condition: the localization error decreases with increasing residual vision in the localization of moving sound sources (*r* = 0.92, *p* < 0.01), while the same is not evident for the localization of static sound sources (*r* = 0.39, *p* = 0.4). Even if a bigger sample of visually impaired participants would be necessary to draw any general conclusion, this result highlights the important role of early visual experience in the development of auditory spatial cognition. The case of low vision children is particularly interesting because it suggests not only that a poor but early visual experience is sufficient to develop the ability to localize static and moving auditory sound sources, but also that degraded vision might represents the condition in which compensation mechanisms manifest.

## Discussion

The acquisition of spatial hearing is of fundamental importance for visually impaired children, because it constitutes a good indicator of the ability to independently navigate in the environment and the propensity to engage in positive social interaction with peers. Indeed while for sighted individuals the visual feedback represents the most important incentive for actions and thus for the development of mobility and social skills (Gori et al., 2016), visually impaired individuals strongly rely on auditory landmarks to encode spatial and social information. To our knowledge, to date there have been no previous studies on spatial hearing in young visually impaired children that directly assessed the role of residual vision on the development of auditory localization in static and dynamic conditions. The present experiment aimed at assessing the effects of total versus partial early visual deprivation on the development of auditory spatial localization abilities in childhood. By comparing the performance of totally blind and low vision children in static and dynamic auditory localization conditions, we demonstrated that visual experience is fundamental for the development of specific auditory spatial skills. Indeed, while total blindness from birth strongly compromises the ability to localize both kinds of sound sources, a congenital but not total visual impairments leads to compensatory mechanisms that allow the individual to correctly perceive the position of static and moving sound sources in the surrounding space. These results reveal that vision has a pivotal role in guiding the maturation of space cognition in the brain even when visual acuity is poor, as indicated by the case of low vision children, and suggest that visual calibration of spatial perception in the first years of life is crucial for normal spatial inference ability.

Scientific research on the development of auditory localization skills in visually impaired children has provided contrasting results, not only because spatial hearing has been studied within the framework of broader research on the cognitive and motor skills development (Warren, 1977; Hueg et al., 2014) but also because the impact of blindness severity was not always primarily considered. Indeed it has been demonstrated that the onset of blindness has a strong impact on spatial performance in adulthood: for example, late blind individuals who lost vision later in life after a normal visual experience during the first years of life perform equally or even better than sighted participants when asked to identify sound sources in horizontal space (Abel et al., 2002; Cappagli et al., 2015; Finocchietti et al., 2015), to determine the relative distance of two sounds presented in far-auditory space (Voss et al., 2004) and to focus auditory attention in the periphery (Fieger et al., 2006). While several hypotheses have been advanced to explain the superior auditory spatial performance of late blind individuals, like the effect of practice with auditory cues (Cappagli et al., 2015), very little is known about the effects of residual vision on the spatial performance in early childhood.

One of the main issues could have been that studies performed on visually impaired children under 3 years of age do not employ psychophysical procedures but they frequently use the sound of familiar voices or toys to gather information about auditory localization abilities in blind children (Hueg et al., 2014). For example, while studies on older children with visual disabilities demonstrated that they have a good spatial hearing in terms of horizontal and vertical sound localization (Ashmead et al., 1998) but a worse performance in more complex auditory tasks (Cappagli et al., 2015; Cappagli and Gori, 2016; Vercillo et al., 2016), studies on infants with severe congenital blindness indicated that they have a developmental delay in sound localization abilities (Fraiberg, 1977) and motor responses to sound (Fraiberg et al., 1966; Adelson and Fraiberg, 1974) but also that they can compensate for the lack of vision with good manipulatory and walking skills which allow the exploration of sounding objects in the environment (Fazzi et al., 2011).

Although it is difficult to compare the studies that investigate the effect of vision loss on auditory spatial skills because they use different methodologies, an important aspect such as the onset of the visual impairment has not always been considered when investigating auditory localization skills in blind individuals. For example, many studies mixed data from children with no visual experience with those of children with partial visual experience in the first period of life (Ashmead et al., 1998). Our study aimed to compare spatial localization skills of totally blind and low vision young children in two tasks assessing the ability to encode static and dynamic sound sources separately. Our study led to two main findings: first, we showed that early visual experience has an important role in the development of spatial cognition, since totally blind children performed overall more poorly than sighted and low vision in all spatial tasks performed; as a second, we pointed out that visual calibration of spatial perception in the first years of life is crucial for the development of normal auditory spatial representation even if vision is degraded, since low vision children performed equally to or better than sighted children, respectively, in the static and dynamic auditory spatial tasks. Further studies will be necessary to further confirm the relevance of our results, since our sample comprised only two congenitally blind children due to the difficult nature of recruiting young children with a total visual deficit. Overall these results shed light on the importance of early assessment of spatial impairments in visually impaired children and early intervention to prevent the risk of isolation and social exclusion. Indeed, it has been shown that early blind children have difficulties not only in auditory and haptic spatial skills (Marmor, 1977; Pasqualotto and Newell, 2007; Röder et al., 2007; Gori et al., 2010; Cappagli et al., 2015) as well as locomotor and mobility skills (Sampaio et al., 1989; Stack et al., 1989; Bigelow, 1992; Perez-Pereira and Conti-Ramsden, 2001), but also in engaging in positive social interaction (Sacks and Kekelis, 1992; Sacks et al., 1992; Rettig, 1994; Guralnick et al., 1996a,b,c; McConnell and Odom, 1999). For this reason, the early assessment of spatial abilities in visually impaired children is fundamental to develop adequate intervention programs to restore or rehabilitate impaired aspects of spatial perception.

## Ethics Statement

The study was approved by the ethics committee of the local health service (ASL3 3 Genovese) and parental or adult informed written consent for the study was obtained in all cases before testing the children.

## Author Contributions

GC collected and analyzed all the data, wrote the article and provided an explanatory hypothesis. EC helped recruiting and motivating visually impaired children. SF helped programming the experiments. MG provided support throughout the study.

## Conflict of Interest Statement

The authors declare that the research was conducted in the absence of any commercial or financial relationships that could be construed as a potential conflict of interest. The reviewer DM and the handling Editor declared their shared affiliation, and the handling Editor states that the process nevertheless met the standards of a fair and objective review.
